# Single Center Retrospective Review of Post-laparotomy CT Abdomen and Pelvis Findings and Trends

**DOI:** 10.3389/fradi.2022.850911

**Published:** 2022-03-04

**Authors:** Dylan C. Steffey, Emad A. Chishti, Maximo J. Acevedo, Luis F. Acosta, James T. Lee

**Affiliations:** ^1^University of Kentucky College of Medicine, Lexington, KY, United States; ^2^Department of Radiology, University of Kentucky College of Medicine, Lexington, KY, United States

**Keywords:** laparotomy, trauma, computed tomography, blunt injury, penetrating injury, damage control laparotomy (DCL)

## Abstract

**Purpose:**

To identify common findings visualized on CT following damage control laparotomy, including post-surgical changes and additional injuries, and to determine change in frequency of post-laparotomy CT at our institution over time.

**Methods:**

Single institution, IRB-Exempt, retrospective review of the University of Kentucky trauma registry from 1/2006 to 2/2019 for all trauma patients undergoing exploratory laparotomy initially and subsequently undergoing CT of the abdomen and pelvis within 24 hours. Operative findings from surgical operation notes and findings reported on post-laparotomy CT were recorded, including vascular and solid organ injuries, operative changes, free intraperitoneal fluid/air, and retroperitoneal findings. Next steps in management were also recorded.

**Results:**

In total 1,047 patients underwent exploratory laparotomy initially at our institution between 1/2006-2/2019. Of those, only 96 had a diagnostic CT of the abdomen and pelvis within 24 h after initial surgery, first occurring in 2010. Among these 96, there were 71 blunt and 25 penetrating injuries. Most common injuries recognized during exploratory laparotomy were bowel/mesentery (55), spleen (34), and liver (26). Regarding CT findings, all patients (96/96, 100%) had residual pneumoperitoneum, 84/96 (87.5%) had residual hemoperitoneum, 36/96 (37.5%) noted post-surgical changes or additional injuries to the spleen, 36/96 (37.5%) to the bowel/mesentery, and 32/96 (33.3%) to the liver, and 34/96 (35.4%) were noted to have pelvic fractures. After CT, 31/96 (32.3%) went back to the OR for relook laparotomy and additional surgical intervention and 7/96 (7.3%) went to IR for embolization. Most common procedures during relaparotomy involved the bowel (8) and solid organs (6).

**Conclusions:**

CT examination within 24 h post damage control laparotomy was exceedingly rare at our institution prior to 2012 but has steadily increased. Frequency now averages 20.5% yearly. Damage control laparotomy is an uncommon clinical scenario; however, knowledge of frequent injuries and common post-operative changes will aid in radiologist detection of additional injuries helping shape next step management and provide adequate therapy.

## Introduction

According to the Centers for Disease Control and Prevention (CDC) Web-based Injury Statistics Query and Reporting System (WISQARS), traumatic injury is the most common cause of mortality and morbidity in patients aged 1–44. Across all age groups, blunt injury is the most common type of trauma, and accounts for approximately 75% of all injuries. Falls and motor vehicle crashes (MVCs) comprise the majority of blunt injury (1). The National Trauma Data Bank Annual Report from the American College of Surgeons reports that only 10% of all trauma is penetrating in nature, usually secondary to gunshot wounds (GSW) and knife injuries (6 and 4% respectively) (2). Additional forms of traumatic injury include machinery or industrial related injuries, burns, and drowning. Factors that determine the type of trauma individuals experience include age, gender, and area of residence. Younger patients are more likely to have injuries secondary to MVCs or penetrating traumas, while falls represent a much higher percentage of injuries in older Americans. Age and gender are both known risk factors for increased case fatality with males and older adults suffering from a higher case fatality rate (2).

Patients that present to the emergency department following trauma who are hemodynamically unstable, unresponsive to resuscitation, and possess a positive Focused Assessment with Sonography in Trauma (FAST) exam may proceed directly to the operating room for damage control laparotomy (DCL) (3). Damage control laparotomy is not a single surgery but rather a limited exploration and intervention focused on stabilization (4). Operations to be performed during this intervention may include splenectomy, hepatorrhaphy, cauterization, vessel ligation, bowel resection, abdominal packing and placement of a temporary closure devise. The goal of this operative technique is to first control active hemorrhage and second to limit contamination (5, 6). Additionally, packing may be retained to further reduce hemorrhage and external drains may be placed to divert contaminants from bowel, biliary, pancreatic, or urinary bladder leaks. By intervening in this nature, the “lethal triad” of hypothermia, acidosis, and coagulopathy may be prevented or interrupted (7). While this intervention has been shown to improve mortality in trauma patients, it is not considered definitive, and many patients will require further intervention (8). Patient's frequently leave the operating room with temporary abdominal closure devices and are transported to the ICU for further resuscitation and medical management (9). It should be noted that the alternative can also be true – a portion of patients will reach maximal benefit from DCL and undergo fascial closure at the time of the primary procedure (10). Factors which may dictate limited exploration vs. complete repair include injury type, anticipated surgical time, and the development of hypothermia or coagulopathy (11). For patients with open abdomens requiring delayed, definitive repair, this is usually planned for 24–72 h following the primary procedure or as earliest as feasible (12).

In patients with less severe physiology upon presentation, (hemodynamically stable, negative FAST), the colloquially described “pan scan,” consisting of multi-detector computed tomography (MDCT) scan of the head, neck, chest, abdomen and pelvis with intravenous contrast, is an essential element in the early evaluation and clinical decision-making algorithm (13). However, neither in the surgical nor radiological literature is there a universally accepted role for MDCT in the 24-h period following life sustaining DCL (14). Given the extent of their injuries, it is unfeasible for these critically ill, but temporarily stable patients to undergo a CT scan. Thus, it would be beneficial to have clearly defined criteria delineating which patients would likely benefit from post-DCL CT (15). Possible scenarios where post-DCL CT would be beneficial include those where an ICU patient remains hemodynamically unstable despite adequate resuscitation, those with continued resuscitative or vasopressor needs, or those with worsening coagulopathies. Additionally, patients with injuries that extend outside the surgical field or in patients where there is a high suspicion for retained foreign materials may also benefit. Lastly, in situations where definitive repair must be delayed or there is concern about the structural integrity of the surgical repair, post-operative CT imaging may aid the care team. While a consensus on patient selection criteria has yet to be determined, MDCT has seen increasing usage in the immediate post-operative period as an adjunct to DCL for further characterization of injuries and to assist with definitive repair planning and medical management (16).

Limited data is currently available on the ideal CT protocol when imaging post-DCL trauma patients. However, acquisition of arterial and venous portal phases images extending from the thoracic inlet to the femoral trochanters optimizes evaluation and characterization of vascular and parenchymal trauma in these patients (17–19). This can be done by acquiring images at 30 second (arterial) and 90 second (portal venous) intervals after administering a single intravenous (IV) bolus of contrast (20). Alternatively, to reduce radiation, a split-bolus technique consisting of delivering two separate boluses of contrast allows the capture of arterial phase and portal venous phase imaging simultaneously during a single CT acquisition cycle (21). The inclusion of a non-enhanced imaging phase can be of benefit to the radiologist, as it allows distinction of active contrast extravasation (hemorrhage) and retained high attenuating post-surgical material such as packing materials, iodinated gauze, sutures, and other foreign bodies. Additional imaging phases and techniques such as the use of oral contrast, delayed urographic phases and CT cystography should be tailored to the patient's specific clinical scenario and suspected injuries (15). The use of oral contrast in general trauma imaging has been shown to be unnecessary and often delays image acquisition (22, 23). However, while there is a lack of literature discussing the use of oral contrast in post-DCL patients, studies have shown benefit of oral contrast in patients suspected of having post-operative bowel leaks, enteric fistulas, and abscesses when there is adequate time for the contrast material to pass through the small bowel (24). Thus, the use of oral contrast in post DCL imaging may be of benefit in specific clinical scenarios where there is a high degree of suspicion for the above pathologies.

A wide range of possible findings, both iatrogenic and non-iatrogenic need to be considered on post-DCL CT imaging. Anatomic alterations such as bowel discontinuity and complete or partial organ removal in the case splenectomy or partial hepatectomy are common findings. Injuries with complete or incomplete surgical repair must also be described. Retained foreign bodies, such as bullets in penetrating trauma or iatrogenically introduced foreign bodies such as hemostatic packing, surgical drains, and temporary abdominal closure devices can be present. Additionally, the physiological changes related to the shock phenomenon such narrowed vessel caliber, edema, and glandular hyperenhancement can all be present. Baghdanian et al. and Alexander et al. have previously described the radiologic appearance of common findings present on post-DCL MDCT in superb detail (14, 17). Ultimately, the role of the radiologist is to identify and distinguish between the multitude of injuries and anatomic alterations related to surgical intervention from those injuries that warrant further surgical repair or those that may be better managed by interventional radiology (IR).

As post DCL-CT examinations are being utilized with increasing frequency, it is important to evaluate and understand both the strengths and weaknesses of this modality. Our study examines utilization trends of post DCL-CT at a large urban ACS Level 1 tertiary care hospital system from 2006 until 2019 and compares the CT findings reported at the time of image acquisition to those noted intraoperatively. Additionally, next steps in patient care were also subsequently categorized and delineated for further analysis.

## Materials and Methods

This study is a single institution, IRB-exempt, retrospective review of the University of Kentucky trauma registry from 1/2006 to 2/2019. The University of Kentucky hospital is an American College of Surgeons-Committee on Trauma accredited Level 1 trauma center servicing as the only tertiary trauma center for the 49 Appalachian counties comprising eastern Kentucky and the 11 non-Appalachian counties surrounding Fayette County (where the University of Kentucky is located). All trauma patients greater than 16 years of age undergoing DCL and who subsequently underwent CT of the abdomen and pelvis within 24 h were included. Patients who did not have a CT scan of the abdomen and pelvis or who were outside the 24-h window were excluded. Given the retrospective nature of this study, informed patient consent was waived. Time to operating room and time to CT were recorded. Mechanism of injury was obtained from history and physical notes completed at time at presentation. Operative findings from surgical procedure notes were recorded, as well as the findings reported on post-DCL CT including vascular and solid organ injuries, operative changes, free intraperitoneal fluid/air, and retroperitoneal findings. Next steps in management such as procedures performed by IR, subsequent surgical operations, and definitive closure were noted. Post–DCL CT findings were compared to the findings noted in the operative reports.

Within this study, we determined the utilization frequency of post-DCL CT over a 14-year period and performed linear regression of the data to assess how utilization has changed. IBM® SPSS® Statistics and Microsoft Excel were used to assess the frequencies of mechanism of injury, operative report findings, radiologic reported findings, operative procedures performed, and subsequent next steps in management. This software was also used to generate descriptive statistics of time from door to DCL (e.g. mean, max, min, SD).

## Results

### Patient Characteristics

**Figure 1:**
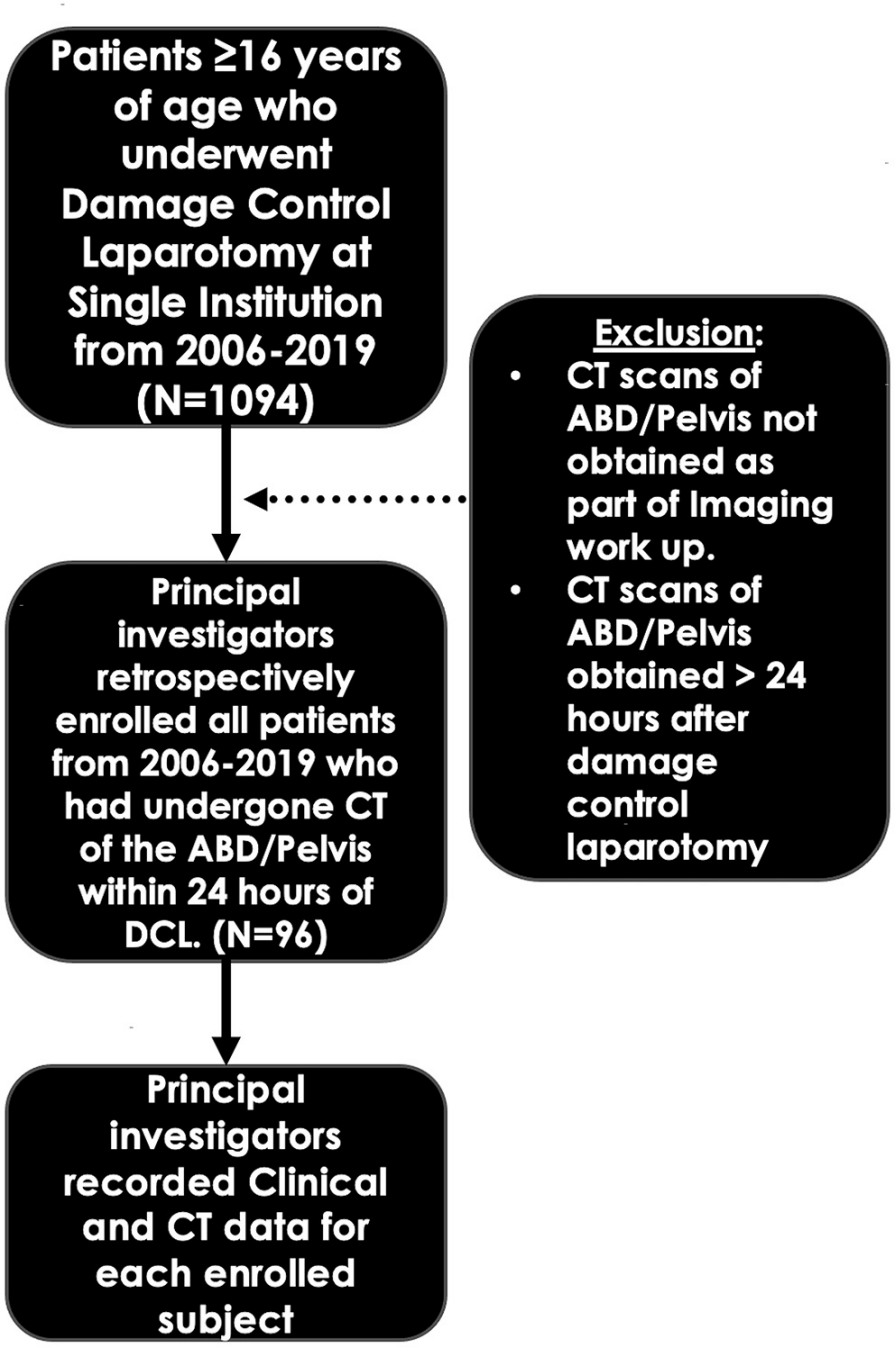
Patient selection algorithm.

Among 1,074 patients who underwent emergent DCL in our cohort from January 2006 until February 2019, only 96 underwent diagnostic CT of the abdomen and pelvis within 24 h of initial surgery. The first recorded patient meeting these criteria was from 2010. Among these 96 patients, 71 sustained blunt injuries while 25 sustained penetrating (gunshot wounds and knife injuries). Other statistics reported from this data bank are also anecdotally consistent with the demographics and characteristics of trauma patients at our institution. The majority of trauma included in our cohort was blunt in nature, specifically from MVCs. Ancillary causes included falls from standing, falls from horseback, and logging injuries, among others. Figure 1 outlines the patient selection algorithm used to determine study inclusion.

### Operative and CT Findings

**Figure 2:**
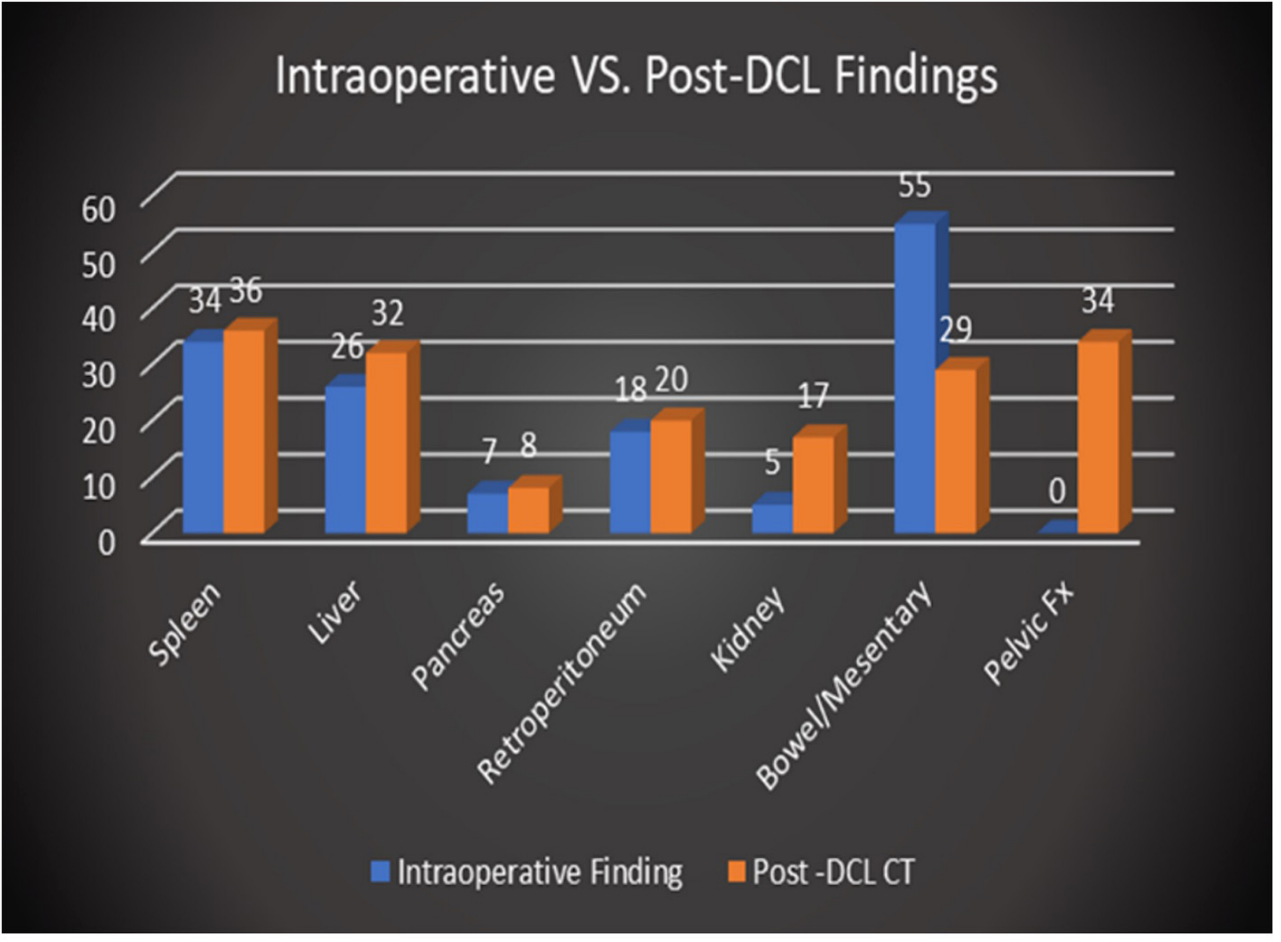
Comparison of overall distribution of injuries reported intraoperatively vs. on post-DCL CT.

The spleen was the most injured solid organ based on intraoperative findings reported at the time of the procedure with 34 instances of splenic injury noted. However, bowel and mesenteric injuries were the most common injuries reported overall with 55 instances in total. Notable post-DCL CT findings included residual pneumoperitoneum (96/96 patients, 100%), residual hemoperitoneum (84/96, 87.5%) pelvic fracture (34/96, 35.4%), and post-surgical changes or additional injuries to the spleen (36/96, 37.5%), bowel/mesentery (36/96, 37.5%), and liver (32/96, 33.3%). Post-DCL CT identified the presence of more solid organ injuries or post-surgical changes (36 vs. 34) as well as more injuries to the kidneys and pelvis (17 vs. 5 and 34 vs. 0, respectively) than those reported in the operative reports. The presence of pelvic fractures and retroperitoneal injuries such as those to the renal system were almost exclusively visualized by imaging given the typical confinement of DCL to the peritoneal space. However, a few retroperitoneal injuries, primarily hematoma formation, were noted intraoperatively. This was largely related to visualization of the injuries through the retroperitoneal wall or via discontinuities of the retroperitoneum. DCL is clearly less sensitive at identifying retroperitoneal injuries, particularly those involving solid organs, than MDCT. Post-DCL CT however, was less effective at describing bowel and mesenteric injuries than DCL with only 29 instances noted on CT reports vs. 55 seen intraoperatively. These injuries, while frequently requiring repair during relook laparotomy, were often small in nature while larger more severe injuries such as perforations and discontinuities were visible. Figure 2 demonstrates the overall distribution of injuries detailed in operative reports in comparison to those found on CT. Severity characterization based on imaging findings was not recorded.

### Next Steps in Management

**Figure 3:**
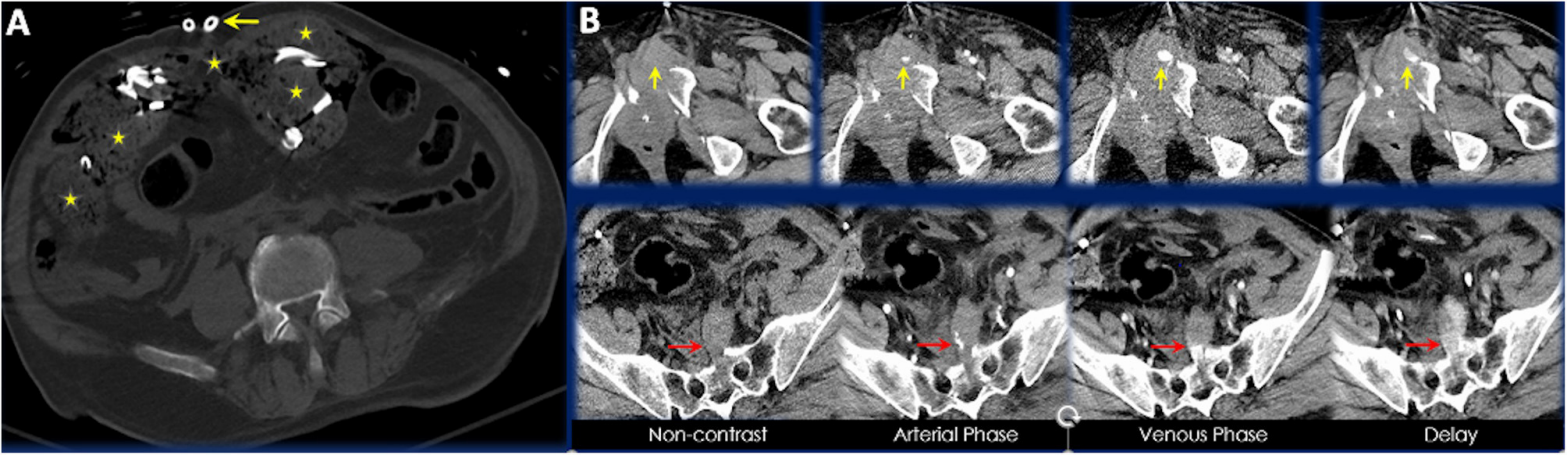
**(A)** Axial CT image of a 70-year-old male presenting as a trauma alert red following motorcycle accident. Placement of surgical drains, retained laparotomy sponges, and preperitoneal hematoma are visible in this axial slice. **(B)** Axial CT images highlighting areas of contrast extravasation in different CT phases. Yellow arrows point to extravasation anterior to an acute comminuted left superior pubic ramus fracture and red arrows point to extravasation anterior to an acute comminuted sacral fracture.

A total of 7 patients (7/96, 7.3%) required intervention by IR following DCL. All 7 patients were blunt trauma victims secondary to MVCs and had pelvic fractures. These pelvic fractures were associated with hemorrhage requiring embolization. Vessels embolized included inferior epigastric, hypogastric, lumbosacral, superior gluteal and others. There lacked any clear trends in injured vessels or types of pelvic fractures within these 7 patients. It should be noted that 4 patients underwent post-DCL CT prior to embolization while the remaining 3 underwent post-DCL CT after embolization (however this was still within 24 h of initial DCL). Of the 4 who underwent post-DCL CT prior to IR intervention, the vascular injury requiring embolization was visualized by CT in 3 of the patients. In the remaining patient, extensive visceral bleeding was present on CT, however pelvic vascular extravasation was only noted during the second operative procedure before transfer to the IR service. Figures 3A,B demonstrates the post-DCL CT findings of an elderly patient following MVC with active extravasation of contrast material necessitating IR intervention for angioembolization.

**Figure 4:**
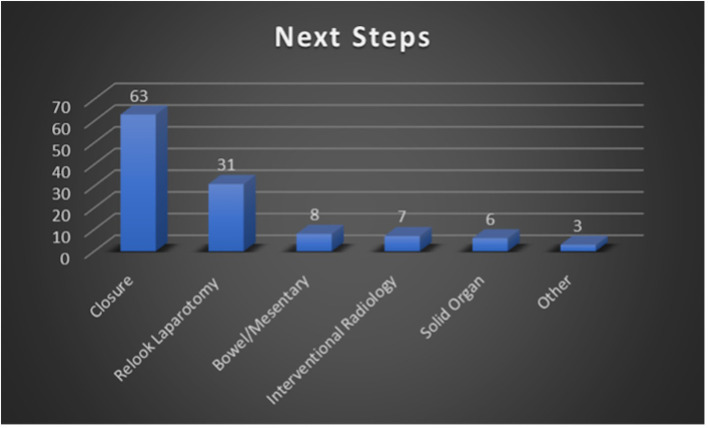
Summary of next step management performed following post-DCL CT. These categories are non-exclusive (e.g., one patient could have undergone both surgical and IR intervention).

Alternatively, 63 patients within our cohort did not require IR intervention and either underwent closure during the primary DCL or underwent delayed closure without further unplanned intraoperative procedures. This would include patients who were packed to achieve hemostasis and then closed later, as well as patients who were left with discontinuity of the bowels with planned, delayed re-anastomosis. A total of 31 patients (31/96, 32.3%) from our cohort required relook laparotomy and additional surgical intervention. The purpose of additional surgical intervention included searching for additional injuries in patients that remained unstable in the ICU, procedures focused on the bowels/mesentery, vascular repair, and additional solid organ procedures. Figure 4 summarizes the quantitative breakdown of next step interventions. It should be noted that these categories are non-exclusive and that some patients may have had multiple interventions. In total, 5 patients required both additional surgical intervention as well as IR embolization.

Additionally, not all injuries reported on post-DCL CT exam necessitated surgical intervention. For example, one patient, a 31-year-old male, presented as a trauma alert red following a high speed MVC with partial ejection. During DCL, the patient underwent small bowel resection secondary to distal ileal injuries. Other intraoperative findings included retroperitoneal hematoma, however there was no mention of a liver laceration in the operative report but was visible on post-DCL CT. Despite the presence of this injury on imaging, the patient did not require any further intra-abdominal procedures.

### Utilization Trends and Statistics

**Figure 5:**
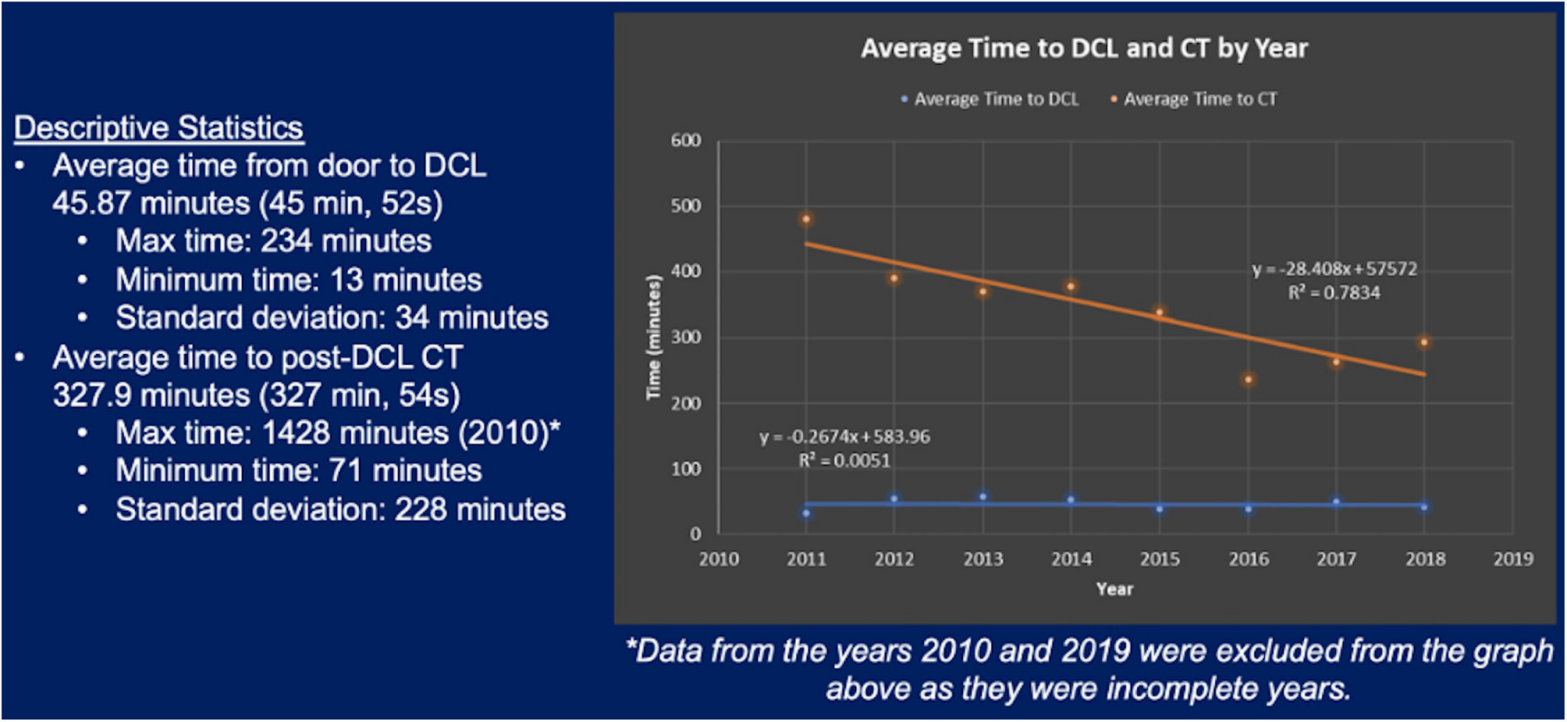
Average time from door to DCL and average time to post-DCL CT between January 2011 and December 2018.

**Figure 6:**
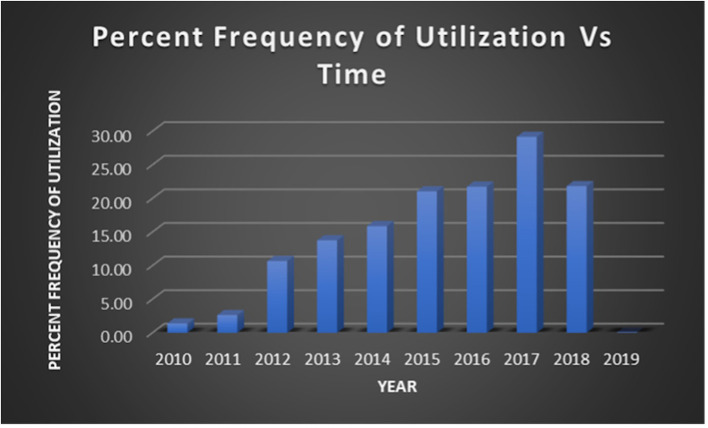
Utilization of post-DCL CT over time from January 2010 to February 2019.

At our institution, the average time from door to DCL was 45 min and 52 seconds with a standard deviation of 34 min. Times ranged from 13 min at minimum to 234 min at maximum. Average time to DCL has remained largely consistent over the years of our study. The first recorded use of post-DCL CT was in 2010. The average time to post-DCL CT was 327 min and 54 seconds with a standard deviation of 228 min across all the years of the study. This average time was noted to have significantly decreased over the study period. Maximum time to post-DCL CT was 1428 min. These data regarding time from door to DCL and time to post-DCL CT are summarized in Figure 5. Over the years, there has also been a steady increase in utilization of post-DCL CT. Peak utilization occurred in 2017 with almost 30% of DCL patients undergoing CT of the abdomen and pelvis within 24 h. Furthermore, average utilization has been consistently above 20% from 2015 through 2018. Data accumulated from calendar year 2019 was limited, therefore assessments regarding utilization trends could not be made. Figure 6 highlights the trends in utilization over the study period.

## Discussion

A clear, positive trend in the use of MDCT as a clinical adjunct to DCL in the immediate post-operative period over the last few years was observed. Furthermore, as prehospital resuscitative and life sustaining capabilities continue to improve, more trauma patients will present to emergency departments in need of immediate intervention. Thus, despite being a relatively uncommon clinical scenario, post-DCL CT will continue to be a part of the radiologist's clinical practice. Baghdanian et al. and Alexander et al. have both published on radiographic findings radiologists should be aware of on post-DCL CT (14, 17).

Understanding the common injuries and post-operative appearance associated with DCL is paramount in aiding the radiologist's detection of additional injuries. These additional injuries may be crucial in understanding why a patient is unresponsive to resuscitation efforts or other medical therapy and may also explain other metabolic derangements displayed on laboratory work. Ultimately, the additional injuries found on post-DCL CT help to identify next step management and ensure adequate therapy is provided. When evaluating post-DCL CT, the radiologist must focus their attention on clinically relevant or actionable findings while paying special attention to areas of the body not typically visible intraoperatively, such as the retroperitoneum and the pelvis. Solid organs may be assessed for occult injuries, post-surgical changes, and complications related to intraoperative procedures. Surgically occult injuries to the bowel and mesentery can be difficult to evaluate as the features typically associated with injury on post-DCL CT, such as pneumoperitoneum, bowel wall edema, and hematoma formation, can all be present secondary to surgical intervention. Nonetheless, there is value in noting these findings as it provides a baseline assessment that can be monitored for improvement or degradation on follow up imaging. Not all injuries noted on post-DCL CT require further intervention but may be important to completely assess polytrauma patients as well as record data recording for ACS registered trauma centers.

In our study, a total of 96 patients underwent CT within 24 h of DCL, with 71 (74%) presenting after sustaining blunt injury and 25 (26%) after penetrating trauma. Our cohort is unique in that it was comprised of a larger percentage of patients who sustained penetrating injury compared to our local as well as nationwide incidence of penetrating trauma of 10% (reported by the American College of Surgeons 2016 National Trauma Data Bank Annual Report) (2). Regarding intraoperative findings in our patients, the spleen was the most injured solid organ with a total of 34 instances noted during the study period. Additionally, injury to the spleen was also the most detected post-DCL CT finding with a total of 36 instances. These findings are unsurprising as the spleen is the most injured organ after blunt trauma and that, in recent years, vascular injuries to the spleen related to blunt trauma are being identified at increasingly higher rates (25, 26). The most common intraoperative finding, however, was injury to the bowel/mesentery with a total of 55 instances. Despite this, injury to the bowel/mesentery on post-DCL CT was reported with lower frequency at a total of 29 instances, which may reflect surgical repair during DCL. With regards to next steps in management following post-DCL CT, 63 patients (66%) required no further surgical intervention, 31 (32%) required additional surgical intervention, and 7 (7.3%) required IR angioembolization as shown by Figure 4 (note that the categories described here and in the figure are non-exclusive). Those that required additional surgical intervention included patients who had planned relook laparotomy or removal of packing material and delayed abdominal wall closures.

We also examined changes in post-DCL CT utilization and trends in usage timeframes at our institution from January 2006 – February 2019. Prior to 2010, no patients at our institution underwent post-DCL CT within 24 h of initial operation. As shown by Figure 6, utilization increased every year from 2010-2017 however dropped slightly in 2018 (2010 and 2019 are limited as data was not obtained from the full year). Utilization was ≤10% from 2010-2012 and ≥20% from 2015-2018. Peak utilization occurred in 2017 with nearly 30% of DCL patients undergoing post-DCL CT. Average time to DCL remained consistent throughout the study period while the average time to post-DCL CT demonstrated a negative trend as indicated by linear-regression (from ~500 min to ~250 min, approximately; Figure 5).

Our study had a few limitations. First, the observational, retrospective study design limits patient selection and potential confounding circumstances. Given the retrospective design, it is difficult to definitively conclude how post-DCL CT findings impacted further patient management. It is likely that a variety of other factors, such as patient specific considerations (e.g., medical history), institutional resource limitations, and availability of consulting physicians, also played a role. Planned repeat laparotomy is common. Another factor to consider is the variability in management preferences of the different trauma physicians at our institution, which likely changed considerably over the 13-year study period. At our institution, no standard protocol was followed to select patients for post-DCL CT and is largely at the discretion of the trauma surgery physician. Furthermore, radiology reports were used for radiologic diagnosis and interpreting radiologist may not have been blinded to operative report. The original radiology reports created at the time of the study were not read by a single physician, and differences in physician reporting styles were not accounted for. The possibility of confounding also exists as there was no clear method to ascertain the selection criteria for which post-operative patients subsequently underwent CT of the abdomen and pelvis. The patients who did qualify for this study represent less than 10% of the source population thus the limited sample size also must be noted.

## Conclusion

CT examination within 24 h post DCL was exceedingly rare at our institution prior to 2012. Utilization has steadily increased since then, with frequency now averaging 20.5% yearly. Post-DCL CT is an uncommon clinical scenario, representing on average less than 100 cases a year at our institution. Within our cohort, CT was better able to show damage to solid organs both within the peritoneal cavity and in the retroperitoneum not noted in the operative report. Moving forward, re-examination of CT images of our patient population by fellowship trained radiologists, who are blinded to the original report, will allow us to determine if there exist any discrepancies in reporting or interpretation of the images. Additionally, it would be beneficial to establish clear clinical indications for post-DCL CT through further retrospective and prospective study designs.

## Data Availability Statement

The raw data supporting the conclusions of this article will be made available by the authors, without undue reservation.

## Ethics Statement

The studies involving human participants were reviewed and approved by University of Kentucky Internal Review Board. Written informed consent for participation was not required for this study in accordance with the national legislation and the institutional requirements.

## Author Contributions

DS: manuscript formation. EC: manuscript editing. MA: statistical analysis. LA: abstract presentation and image curation. JL: hypothesis formation, study design, research/administration, and manuscript editing. All authors contributed to the article and approved the submitted version.

## Conflict of Interest

The authors declare that the research was conducted in the absence of any commercial or financial relationships that could be construed as a potential conflict of interest.

## Publisher's Note

All claims expressed in this article are solely those of the authors and do not necessarily represent those of their affiliated organizations, or those of the publisher, the editors and the reviewers. Any product that may be evaluated in this article, or claim that may be made by its manufacturer, is not guaranteed or endorsed by the publisher.

## References

[1] Centers for Disease Control Prevention. Web-based Injury Statistics Query and Reporting System (WISQARS). Available online at: https://www.cdc.gov/injury/wisqars/index.html. (accessed February 7, 2022).

[2] Committee Committee on Trauma American American College of Surgeons. National Trauma Data Bank Annual Report 2016. Available online at: https://www.facs.org/-/media/files/quality-programs/trauma/ntdb/ntdb-annual-report-2016.ashx. (accessed February 7, 2022).

[3] Roberts DJ Bobrovitz N Zygun DA Ball CG Kirkpatrick AW Faris PD. Indications for use of thoracic, abdominal, pelvic, and vascular damage control interventions in trauma patients: A content analysis and expert appropriateness rating study. J Trauma Acute Care Surg. (2015) 79:568–79. 10.1097/TA.0000000000000821 26402530

[4] Lee JC Peitzman AB. Damage-control laparotomy. Curr Opin Crit Care. (2006) 12:346–50. 10.1097/01.ccx.0000235213.63988.9a 16810046

[5] Shapiro MB Jenkins DH Schwab CW Rotondo MF. Damage control: collective review. J Trauma. (2000) 49:969–78. 10.1097/00005373-200011000-00033 11086798

[6] Burch JM Ortiz VB Richardson RJ Martin RR Mattox KL Jordan GL. Abbreviated laparotomy and planned reoperation for critically injured patients. Ann Surg. (1992) 215:476–83. 10.1097/00000658-199205000-00010 1616384 PMC1242479

[7] Moore EE. Staged laparotomy for the hypothermia, acidosis, and coagulopathy syndrome. Am J Surg. (1996) 172:405–10. 10.1016/S0002-9610(96)00216-4 8942535

[8] Roberts DJ Bobrovitz N Zygun DA Ball CG Kirkpatrick AW Faris PD. Indications for use of damage control surgery in civilian trauma patients: a content analysis and expert appropriateness rating study. Ann Surg. (2016) 263:1018–27. 10.1097/SLA.0000000000001347 26445471

[9] Parr MJ Alabdi T. Damage control surgery and intensive care. Injury. (2004) 35:713–22. 10.1016/j.injury.2004.03.010 15203312

[10] Hatch QM Osterhout LM Podbielski J Kozar RA Wade CE Holcomb JB. Impact of closure at the first take back: complication burden and potential overutilization of damage control laparotomy. J Trauma. (2011) 71:1503–11. 10.1097/TA.0b013e31823cd78d 22182860

[11] Loftus TJ Efron PA Bala TM Rosenthal MD Croft CA Walters MS. The impact of standardized protocol implementation for surgical damage control and temporary abdominal closure after emergent laparotomy. J Trauma Acute Care Surg. (2019) 86:670–8. 10.1097/TA.0000000000002170 30562327 PMC6433520

[12] Sagraves SG Toschlog EA Rotondo MF. Damage control surgery–the intensivist's role. J Intensive Care Med. (2006) 21:5–16. 10.1177/0885066605282790 16698739

[13] Weis JJ Cunningham KE Forsythe RM Billiar TR Peitzman AB Sperry JL. The importance of empiric abdominal computed tomography after urgent laparotomy for trauma: do they reveal unexpected injuries? Surgery. (2014) 156:979–85. 10.1016/j.surg.2014.06.044 25239355

[14] Alexander LF Hanna TN LeGout JD Roda MS Cernigliaro JG Mittal PK. Multidetector CT findings in the abdomen and pelvis after damage control surgery for acute traumatic injuries. Radiographics. (2019) 39:1183–202. 10.1148/rg.2019180153 31283454

[15] Ahmad ZY Baghdanian AH Baghdanian AA. Multidetector Computed Tomography Imaging of Damage Control Surgery Patients. Radiol Clin North Am. (2019) 57:671–87. 10.1016/j.rcl.2019.02.003 31076025

[16] Boscak AR Shanmuganathan K Mirvis SE Fleiter TR Miller LA Sliker CW. Optimizing trauma multidetector CT protocol for blunt splenic injury: need for arterial and portal venous phase scans. Radiology. (2013) 268:79–88. 10.1148/radiol.13121370 23449955

[17] Baghdanian AH Armetta AS Baghdanian AA LeBedis CA Anderson SW Soto JA. of Major vascular injury in blunt abdominopelvic trauma. Radiographics. (2016) 36:872–90. 10.1148/rg.2016150160 27163596

[18] Holly BP Steenburg SD. Multidetector CT of blunt traumatic venous injuries in the chest, abdomen, and pelvis. Radiographics. (2011) 31:1415–24. 10.1148/rg.315105221 21918052

[19] Miller LA Shanmuganathan K. Multidetector CT evaluation of abdominal trauma. Radiol Clin North Am. (2005) 43:1079–95. 10.1016/j.rcl.2005.08.007 16253663

[20] Beenen LF Sierink JC Kolkman S Nio CY Saltzherr TP Dijkgraaf MG. Split bolus technique in polytrauma: a prospective study on scan protocols for trauma analysis. Acta Radiol. (2015) 56:873–80. 10.1177/0284185114539319 25033993

[21] Stafford RE McGonigal MD Weigelt JA Johnson TJ. Oral contrast solution and computed tomography for blunt abdominal trauma: a randomized study. Arch Surg. (1999) 134:622–6. 10.1001/archsurg.134.6.622 10367871

[22] Clancy TV Ragozzino MW Ramshaw D Churchill MP Covington DL Maxwell JG. Oral contrast is not necessary in the evaluation of blunt abdominal trauma by computed tomography. Am J Surg. (1993) 166:680–4. 10.1016/S0002-9610(05)80679-8 8273849

[23] Stuhlfaut JW Soto JA Lucey BC Ulrich A Rathlev NK Burke PA. Blunt abdominal trauma: performance of CT without oral contrast material. Radiology. (2004) 233:689–94. 10.1148/radiol.2333031972 15516605

[24] Becker CD Gal I Baer HU Vock P. Blunt hepatic trauma in adults: correlation of CT injury grading with outcome. Radiology. (1996) 201:215–20. 10.1148/radiology.201.1.8816546 8816546

[25] Soto JA Anderson SW. Multidetector CT of blunt abdominal trauma. Radiology. (2012) 265:678–93. 10.1148/radiol.12120354 23175542

[26] Lee JT Slade E Uyeda J Steenburg SD Chong ST Tsai R. American society of emergency radiology multicenter blunt splenic trauma study: CT and clinical findings. Radiology. (2021) 299:122–30. 10.1148/radiol.2021202917 33529133 PMC7997613

